# Brain gray matter alterations in Chinese patients with chronic knee osteoarthritis pain based on voxel-based morphometry

**DOI:** 10.1097/MD.0000000000010145

**Published:** 2018-03-23

**Authors:** Xia Liao, Cuiping Mao, Yuan Wang, Qingfeng Zhang, Dongyuan Cao, David A. Seminowicz, Ming Zhang, Xiaoli Yang

**Affiliations:** a Department of Pain; b Department of Nutrition; c Department of Medical Imaging, the First Affiliated Hospital of Xi’an Jiaotong University; d Key Laboratory of Shaanxi Province for Craniofacial Precision Medicine Research, Research Center of Stomatology, Stomatological Hospital, Xi’an, Jiaotong University, Xi’an, Shaanxi, China; e Department of Neural and Pain Sciences, University of Maryland School of Dentistry, Baltimore, Maryland.

**Keywords:** chronic pain, gray matter, knee osteoarthritis, magnetic resonance imaging, voxel-based morphometry

## Abstract

Altered cerebral gray matter volume (GMV) is commonly found in patients with chronic pain. Chronic pain is the prominent characteristic of knee osteoarthritis (KOA), yet little is known about its morphological changes in the brain. Here an MRI study was performed to examine the structural brain abnormalities in 30 KOA patients with knee pain and age-matched healthy subjects. We detected that the patients exhibited significant almost 2-fold age-related decreases of GMV compared to healthy controls. Moreover, KOA patients also had significant loss of regional GMV including in the bilateral orbital frontal cortex (OFC), the right lateral prefrontal cortex (lPFC), and precentral and postcentral cortices. In addition, a high proportion of KOA patients exerted abnormal scores of Hamilton Depression Rating Scale (HAMD), Hamilton Anxiety Scale (HAMA), Mini Mental State examination (MMSE), and Montreal Cognitive Assessment (MoCA) compare to controls. Our results imply that chronic pain conditions which preferentially involve PFC might consider as a “cognitive state.” And emotion and cognitive function about chronic pain should be highly regarded.

## 1 Introduction

Osteoarthritis (OA) is the third most common diagnosis made by general practitioners in older patients and the commonest cause of disability at older ages.^[1]^ It is a chronic joint disease, characterized by articular cartilage degeneration and bone hyperplasia. There is high prevalence in knee pain caused by OA in elderly persons.^[2–5]^ About one quarter of people over the age of 55 experience significant knee pain; half of these have radiographic OA, which is an increasingly common cause of knee pain and a quarter have significant disability.^[6]^ Moreover, 10% of adults aged over 55 report some painful knee osteoarthritis associated disability.^[6]^

Noninvasive brain imaging technologies provide the opportunity to examine brain processes in human clinical pain conditions, and significant progress has been made in this direction. In the past decade, a number of literature reported brain morphological changes in different clinical pain, such as chronic back pain (CBP),^[7,8]^ fibromyalgia,^[9–12]^ complex regional pain syndrome (CRPS),^[13]^ headache,^[14–17]^ irritable bowel syndrome (IBS),^[18,19]^ chronic vulvar pain,^[20]^ and dysmenorrhea.^[21]^ Specifically, Apkarian et al^[7]^ firstly detected gray matter atrophy in patients with chronic low back pain, and found gray matter density reduced in bilateral dorsolateral prefrontal cortex and right thalamus, which suggests that the pathophysiology of chronic pain includes thalamocortical processes. Imaging studies have defined a pain network of somatosensory: primary and secondary somatosensory, insular (S1, S2, IC), limbic: IC, anterior cingulate cortex (ACC) and associative: prefrontal cortex (PFC), which receive parallel inputs from multiple nociceptive pathways, instead of only locating a singular “pain center” in brain.^[22]^ Acute pain in normal participants and chronic clinical pain conditions have distinct but overlapping brain activation patterns.^[22]^ Contrary to the former, chronic pain preferentially involve PFC, and decreased incidence of activity across ACC, S1, S2, IC, and thalamus. It may be suggested that chronic pain tend to lesson sensory processing and increase emotional/cognitive processing.^[22]^

Voxel-based morphometry (VBM) has been widely used to examine region-specific changes in gray matter volume (GMV) in chronic pain patients. Recent studies have begun to explore the brain morphological alterations in OA. Rea Rodriguez-Raecke et al found gray matter decrease in hip OA patients in the ACC, right IC and operculum, dorsolateral prefrontal cortex (DLPFC), amygdala, and brainstem compared with controls. While another report about hip OA found GMV decreased in thalamus.^[23]^ In addition, Baliki et al found that gray matter (GM) density decreases in KOA, which was distinct from CBP and CRPS. However, there are limited studies on GMV abnormalities associated with OA, and little is known about brain morphology in OA, especially in KOA. In the present study, we aimed to determine the GMV differences between KOA patients and matched controls.

## 2 Materials and methods

### 2.1 Participants

This study is a cross-sectional case–control study in China. Thirty patients (4 males and 26 females; age 56.5 ± 6.8 years, mean ± SD) with bilateral KOA were recruited from the outpatient clinic in the department of pain, the First Affiliated Hospital of Xi’an Jiaotong University in China. The patients were only included if they fulfilled criteria of the American College of Rheumatology for classification of OA (Altman 1986) and had no history of other pain conditions. All included patients experienced KOA pain for a duration longer than 3 months with a pain magnitude of at least 3/10 on a visual analog scale (VAS). The patients continued their normal medication usage for pain during the study. Thirty age and sex matched healthy volunteers (4 males and 26 females; age 55.2 ± 5.7 years, mean ± SD) with no history of chronic pain were recruited through advertisement. All patients and controls were screened for the following exclusion criteria: have contraindications for MRI study (e.g., metal implants or claustrophobia); have dentures; or have severe concomitant neurological or psychiatric disorders, or other diseases, such as hypertension, diabetes or coronary disease.

All subjects provided informed consent, and all procedures were performed with permission of the relevant ethics committees, Xi’an Jiaotong University.

### 2.2 Methods

#### 2.2.1 Assessment of clinical pain, cognitive, and affective state

Pain severity was assessed using short-form McGill Pain Questionnaire (SF-MPQ), which includes VAS (0: no pain; 10: maximum imaginable pain) on the scanning day. The duration of pain was measured in years. Affective symptoms and cognitive function were evaluated in all subjects using the following questionnaires: Hamilton Depression Rating Scale (HAMD), Hamilton Anxiety Scale (HAMA), Mini Mental State examination (MMSE), and Montreal Cognitive Assessment (MoCA).

Mood disorder was assessed by HAMD^[24]^ (no depression: 0–7; mild depression: 8–16; moderate depression: 17–23; and severe depression≥24) and HAMA^[25]^ (no anxiety: 0–7; mild anxiety: 8–14; moderate anxiety: 15–21; and severe anxiety≥22). A score of less than 7 was considered normal.

Cognition was evaluated by MMSE^[26]^ and MoCA.^[27]^ In this study, a score of 24 or less was found to be the optimal cut-off point for a diagnosis for cognitive impairment according to MMSE, according to which subjects were divided into 4 ranks: score 0–12 = severe cognitive impairment, score 13–18 = moderate cognitive impairment, score 19–24 = mild cognitive impairment, and score 25–30 = cognitive impairment absent. MoCA scores of 26 or higher were considered normal in terms of cognitive functions.

#### 2.2.2 MRI data acquisition

MRI was performed on a 3.0 T MRI system (General Electric Signa HDXT, Milwaukee, WI) using a three-dimensional T1-weighted fast spoiled gradient echo sequence with the following parameters: repetition time = 10.8 ms, echo time = 4.8 ms, matrix = 256 × 256, field of view = 256 mm × 256 mm, slice thickness = 1 mm, space between slices = 0, 140 axial slices, scan duration = 5 minutes. Routine axial T2-weighted images were analyzed for the presence of organic lesions accompanied by T1-weighted images. All images were visually inspected by two neuroradiologists, and those with excessive motion artifacts were excluded.

#### 2.2.3 MRI data processing

The T1-anatomical brain images were used to calculate the volume of GM, white matter (WM) and cerebrospinal fluid (CSF), with SIENAX, part of FSL 5.0 software (http://www.fmrib.ox.ac.uk/fsl/),^[28]^ which used an automated brain extraction and tissue segmentation algorithm to yield estimates of volumes of interest.^[29,30]^ It first stripped nonbrain tissue, and then used the brain and skull images to estimate the scaling between the subjects’ image and standard space. It then ran tissue segmentation to estimate the volume of brain tissue, and multiplied this by the estimated scaling factor to reduce head-size-related variability between subjects.

Regional GMV was assessed with FSL 5.0 software VBM function (http://www.fmrib.ox.ac.uk/fsl/).^[30–32]^ First, structural images were brain-extracted using BET.^[33]^ Then, tissue-type segmentation was carried out using FAST4.^[34]^ The resulting gray-matter partial volume images were then aligned to MNI152 standard space using the affine registration tool FLIRT,^[35,36]^ followed by nonlinear registration using FNIRT, which used a b-spline representation of the registration warp field. The resulting images were averaged to create a study-specific template, to which the native gray matter images were nonlinearly re-registered. The registered partial volume images were then modulated (to correct for local expansion or contraction) by dividing using the Jacobian of the warp field. The modulated segmented images were then smoothed with an isotropic Gaussian kernel with 8 mm full-width at half maximum (FWHM).

Regional changes in gray matter were assessed using permutation-based inference^[37]^ to allow rigorous cluster-based comparisons of significance within the framework of the general linear model with *P* values less than .05, fully corrected for multiple comparisons. Group differences were tested with 5000 random permutations, which inherently accounted for multiple comparisons. Age, sex, and total intracranial volume (ICV) were all used as covariates of no interest.

#### 2.2.4 Statistical analysis for tissue volumes and questionnaire data

Statistical analysis was performed with SPSS 13.0 software (SPSS, Chicago, IL). Demographic, clinical, and tissue volume (total WMV, GMV, CSF) differences between the groups were tested using Student's *t*-test, χ^2^-test, the Mann–Whitney rank sum test, as appropriate. Results were considered significant at *P < *.05. Two independent-samples *t*-test was used to compare differences between groups for demographic data and brain tissue volume, and χ^2^-test and Mann–Whitney rank sum test for MMSE, MoCA, HAMD, and HAMA data. Spearman rank correlation was used to assess the relationships between GMV and pain duration, pain characteristics and psychometric variables as well. The psychometric data included: the MMSE, MoCA, HAMD, and HAMA scores. For all of these analyses *P < *.05 was considered statistically significant. But to avoid multiple comparisons, adjusted significant level should be performed in this study as appropriate.

## 3 Results

### 3.1 Demographic, painful, and psychometric information on the subjects

Thirty KOA patients and 30 age, sex matched healthy control subjects were included in this study. The MMSE, MoCA scores were significantly lower and the HAMD and HAMA scores were significantly higher in KOA patients than in healthy controls (P < .05) (Table 1). A high proportion of KOA patients exerted abnormal scores for MMSE, MoCA, HAMD, and HAMA compare to controls. Detailed psychometric and demographic data are shown in Table 1.

**Table 1:**
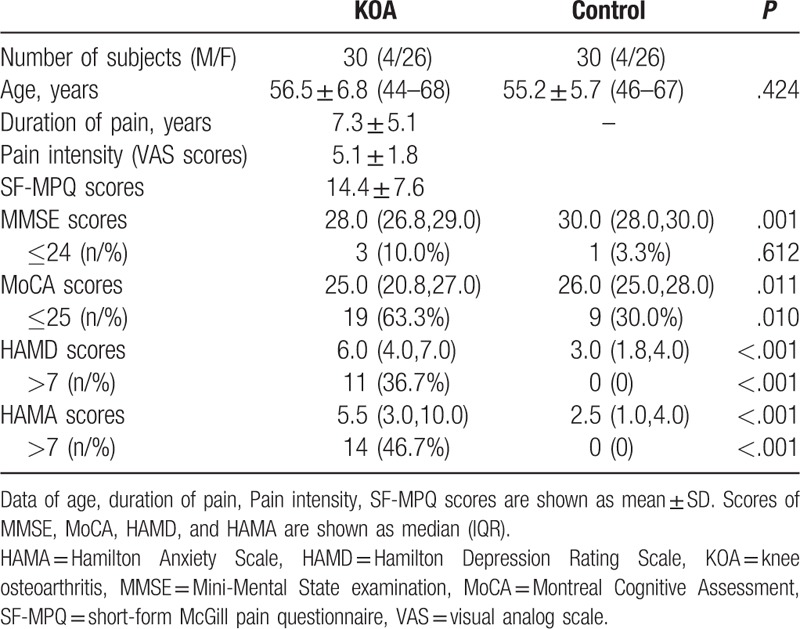
Demographic and clinical data of the participants.

### 3.2 Brain tissue volume

**Figure 1:**
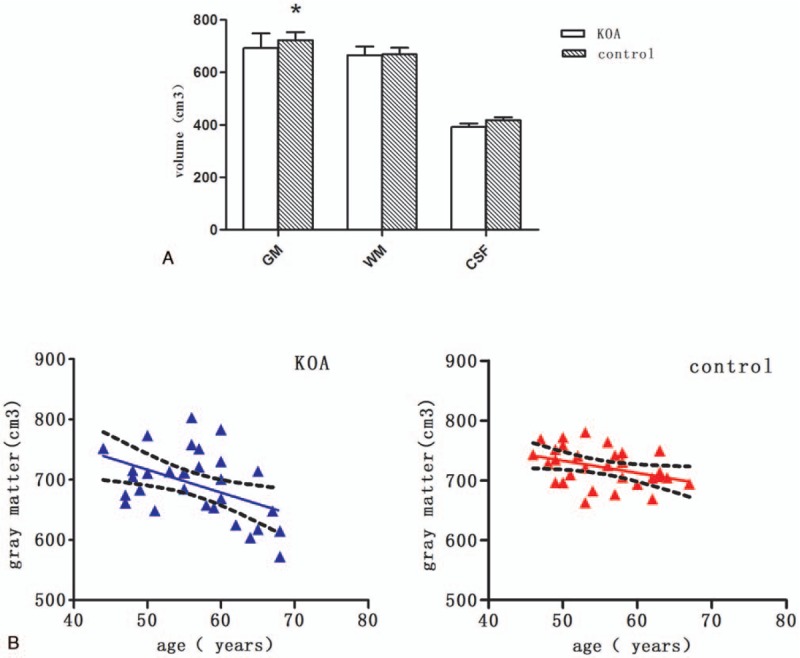
Gray matter and its correlation with age in KOA patients and healthy control subjects. (A) Gray matter, white matter and CSF in KOA patients and healthy control subjects. ^∗^*P < *.05. (B) Gray matter volume was negatively correlated with age in KOA patients (right) and healthy subjects (left). KOA subjects: age dependence slope = –3.8 cm^3^, with *r*^2^ = 0.212; controls: slope = –2.1 cm^3^, with *r*^2^ = 0.145, *P < *.05 in both groups. CSF = cerebrospinal fluid, GM = gray matter, WM = white matter.

As shown in Figure 1A, KOA patients had significant reductions in GMV compared with healthy controls. There was no difference in the WMV and CSF between two groups. In addition, GMV was negatively correlated with age in both KOA patients and healthy controls (KOA subjects, age dependence slope = −3.8 cm^3^, with *r*^2^ = 0.212; controls, slope = −2.1 cm^3^, with *r*^2^ = 0.145, *P < *.05 in both groups, Fig. 1B). The age-associated loss in gray matter in the KOA group was 3.8 cm^3^/year, which was significantly greater than the loss in matched controls (only 2.1 cm^3^/year). We found a negative trend between gray matter and duration, with no statistical significance (Spearman rank test: *r* = −0.144, *P* = .448).

### 3.3 Regional GMV

**Figure 2:**
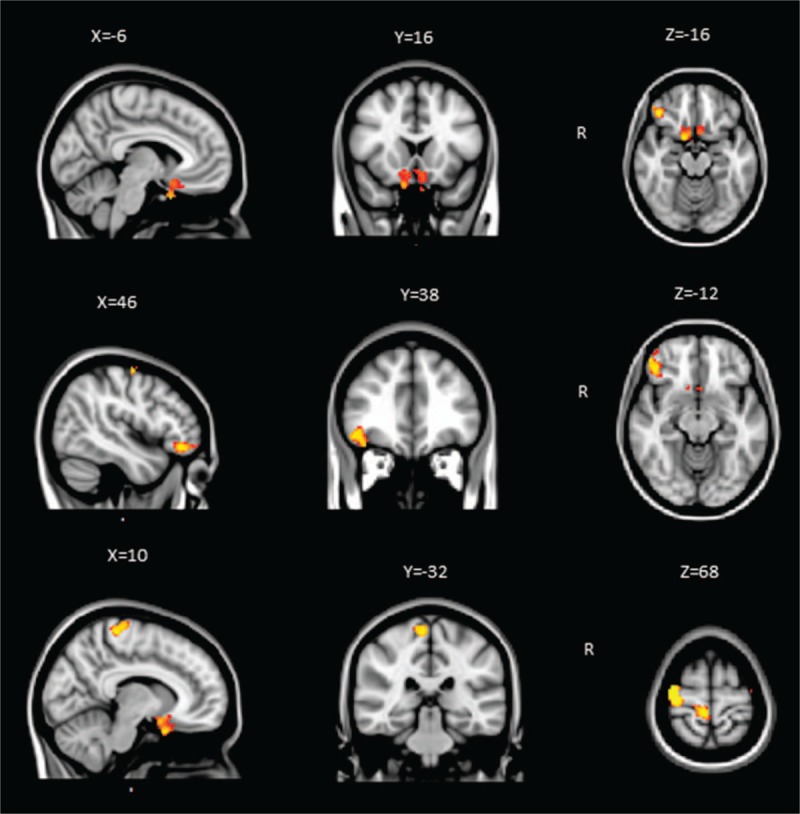
Gray matter volume decreased in several brain areas in patients with KOA. GMV was decreased in the bilateral orbital frontal cortex (OFC), the right lateral prefrontal cortex (lPFC), and the precentral and part of postcentral cortex (*P < *.05, FWE corrected). Between-group differences were represented as statistical maps color-coded on a red-yellow scale, with brighter (more yellow) regions corresponding to more significant differences. Images were presented with right hemispheric structures shown on the right. R: Right. GMV = gray matter volume, KOA = knee osteoarthritis.

The results from whole brain voxel-based morphometry analysis showed that GMV decreased in several cortical structures in KOA patients compared with healthy controls, including the bilateral orbital frontal cortex (OFC), the right lateral prefrontal cortex (lPFC), the precentral and part of postcentral cortex (*P < *.05, FWE corrected; Fig. 2). There are no significant volumetric increases in the any brain regions.

### 3.4 Correlations among affective and cognitive in KOA patients

**Figure 3:**
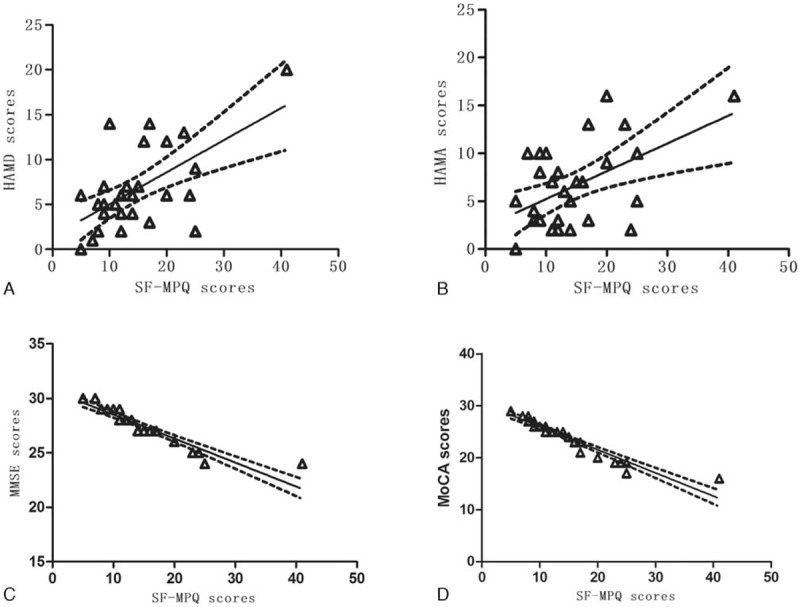
Correlation among psychometric variables in KOA patients (Spearman rank correlation). (A) Positive trend between SF-MPQ scores and HAMD scale scores (*r* = 0.441, *P* = .015); (B) positive trend between SF-MPQ scores and HAMA (*r* = 0.328, *P* = .077); (C) negative correlation between MMSE scores and SF-MPQ scores (*r* = −0.983, *P* < .01); (D) negative correlation between the MoCA scores and SF-MPQ scores (*r* = −0.986, *P* < .01). HAMA = Hamilton Anxiety Scale, HAMD = Hamilton Depression Rating Scale, KOA = knee osteoarthritis, MMSE = Mini Mental State examination, MoCA = Montreal Cognitive Assessment, SF-MPQ = short-form McGill Pain Questionnaire.

In KOA patients, a positive trend was found between pain intensity and HAMD scores (Spearman rank test: *r* = 0.441, *P* = .015, adjusted significant level = 0.0125; Fig. 3A) and HAMA scores (Spearman rank test: *r* = 0.328, *P* = .077, adjusted significant level = 0.0125; Fig. 3B), but with no statistical significance. The pain intensity in KOA patients was negatively correlated with MMSE scores (Spearman rank test: *r* = −0.983, *P < *.01, adjusted significant level = 0.0125; Fig. 3C) and MoCA scores (Spearman rank test: *r* = −0.986, *P < *.01, adjusted significant level = 0.0125; Fig. 3D).

## 4 Discussion

### 4.1 KOA patients have abnormal loss of gray matter

The present study revealed that patients with KOA have atrophy of brain gray matter. Interestingly, the age-related decrease in GMV accelerated in KOA patients. The age associated decrease in neocortical GMV was 3.8 cm^3^ per year in KOA group, which is almost two times as that of age-matched healthy subjects. These changes were similar to the previous estimations of age-dependent gray matter atrophy.^[7,10,13,31,38]^ In chronic low back pain^[7]^ and fibromyalgia patients,^[10]^ the magnitude of brain gray matter atrophy is, respectively, equivalent to1.2 years and 9.5 years of aging in normal population.

There is strong evidence that gray matter atrophy is age dependent and this is supported by cross-species postmortem data.^[39,40]^ We found that gray matter in KOA patients presented higher shrink than normal aging. Gray matter decreases faster in different types of chronic pain compare with normal human. Some researchers discussed this phenomenon as atrophy,^[7,10,14,41]^ which is sustained by the fact of significant correlation between brain gray matter alterations and duration of pain.^[42]^ Gray matter loss, such as described in the patients with chronic pain, may be attributed to the destruction of the neurons and cells atrophy or the loss of synapses, and decreases in cell size or blood volume may also accounted for these changes.^[43,44]^ In fact, histopathology is the gold standard method, explaining the mechanism of structural changes. However, histological data are lacking, especially lack of direct histological evidence for global or regional brain atrophy in humans with chronic pain.^[15]^ It is should be emphasized that in some longitudinal studies,^[23,45,46]^ suggested that neurodegenerative process with irreversible damage is impossible.^[47]^

### 4.2 Regional gray matter atrophy

In this present study, we found that gray matter decreased in the bilateral orbital frontal cortex (OFC), the right lateral prefrontal cortex (lPFC), the precentral and part of postcentral cortex.

A large number of studies show that chronic pain is associated with gray matter loss, although the specific regions involved vary in different syndromes, which suggests these distinct diseases have potential mechanism of brain atrophy, and the regional differences of gray matter reduction could be the explanation of discrepancies between symptoms.^[10]^ PFC has extensive connections with other cortical areas and subcortical nuclei, and plays an important role in execution, attention, memory, and evaluation. It is strongly links with mood and cognitive function. In the present study, gray matter decline in PFC is consistent with those from the previous studies.^[23,45,48,49]^ Spontaneous chronic pain is considered an emotional state, as well as in OA pain.^[48,49]^ Moreover, brain regions involved in spontaneous pain may distort assessment and prediction of outcomes based on emotional guide,^[37,50–52]^ which may be the reason of decision-making obstacles in different chronic pain conditions,^[53]^ and the similar obstacles could be observed in KOA patients. While medial prefrontal-limbic cortical areas are always engaged spontaneous pain, different brain areas might be activated in varied clinical states, such as involvement of medial prefrontal cortex (mPFC) in chronic back pain^[54]^ and amygdala and accumbens by postherpetic neuralgia.^[55]^ Parks et al^[49]^ found that more orbitofrontal cortical regions were involved in KOA using fMRI combined with psychophysics, with orbital, medial, and lateral PFC activity reflecting its primary clinical characteristics. In the present study, we found decreased GMV in OFC and lPFC. A large number of animal and clinical imaging studies^[56–59]^ suggest that cingulate-prefrontal cortex (including rostral cingulate and OFC) is related with processing and modulation of pain. Cingulate-prefrontal cortex also plays an important role in cognition and mood of pain with interactions of ascending and descending pathways.

In the previous fMRI studies, activity in motor and somatosensory cortex (precentral and postcentral cortex) was mostly found in the evoked pain condition.^[49,60,61]^ Parks et al considered that activated brain regions were significant different between stimulus-evoked pain and spontaneous pain, since the regions involved in stimulus-evoked pain were commonly observed in acute pain state.^[22,49,62]^ However, Kulkarni et al^[48]^ found motor and somatosensory cortex were activated in both spontaneous pain and pain-free conditions in KOA patients. The inconsistency of these results may be associated with technology and sample heterogeneity. In addition, it seems to exude an increase in gray matter in somatosensory areas for chronic pain patients suffered constant pain. This lack of gray matter increase in somatosensory areas may due to absence of a significant noxious input.^[45]^

### 4.3 Affective and cognitive variables in KOA patients

In our study, there were higher percentage persons with abnormal scores in MMSE, MoCA, HAMD, and HAMA in KOA patients than controls. It was observed that scores evaluated emotion and cognitive function are related to severity of pain.

Chronic pain not only manifests an unpleasant feeling in physiology, but also complex psychological emotions affected by society and environment. Chronic pain is generally associated with negative emotions. Katz and colleagues considered that postherpetic neuralgia patients with long-term pain (duration more than 3 months) always accompanied by severe depressive symptoms compared with pain-free patients.^[63]^ Meanwhile, Demyttenaere et al^[64]^ estimated emotional abnormalities of chronic pain patients in 17 countries worldwide, and found the incidence rate of emotional disorders was 10% to 42%, mainly including anxiety and depression. KOA patients generally have the depressive symptoms, which may be associated with increased pain and development of joint damage.^[65]^ In addition, in an fMRI study of 20 healthy people, when participants had depressed mood, pain perception enhanced, namely, pain strengthen itself with unpleasant emotional experience.^[66]^ Depression and pain disorders are common comorbidities. Common neurocircuitries (e.g., the hypothalamicpituitary-adrenal axis, limbic and paralimbic structures, ascending and descending pain tracks) and neurochemicals (e.g., monoamines, cytokines, and neurotrophic factors) play an important role correlating the pathophysiologies of depression and pain disorders. Changes in neurocircuitries and neurochemicals caused by one disorder can affect another disorder.^[67]^ However, the underlying relationship between chronic pain and depression remains unknown,^[68]^ with some emerging evidence that pain is precedential.^[69]^

Cognitive complaints are frequently reported by patients with chronic pain that impair social situations and daily life activities.^[70,71]^ Several studies have found that lots of persons with chronic pain complained decrease of memory and concentration.^[72,73]^ Previous researches in term of chronic pain and cognition showed mild-to-moderate deficit in executive functional performance,^[74]^ implied that chronic pain patients often reveal memory impairment.^[75]^ The exact mechanism underlying the complex relationship between pain, memory, and attention is not fully understood; however, it is known that the same neural networks used for many cognitive functions are also used for nociceptive functions.^[76]^

Another important aspect is that many chronic pain patients suffer from asthenia, treatment side effects, sleeplessness as well as depression/anxiety, stress, or a combination of those, which influences the cognitive functions directly or indirectly. Hae Jin Ko et al discussed that patients with depressive symptoms have more subjective memory complaints.^[77]^ Therefore, pain may not be the independent factor affected cognition, and cognitive function also affected by other clinical characteristics (e.g., depression) which is a component of chronic pain.

## 5 Limitations

The present study has some limitations that need further consideration. First, this study is a cross-sectional study. A single point MRI cannot provide brain dynamic and temporal changes. Second, our study has small sample sizes, which might affect reliability of results. Third, females on average have an earlier age of onset of KOA than males, and the sample in the present study was predominantly female. Therefore, the sex differences have to be interpreted with caution, and further well-matched studies are required to confirm the study findings. In addition, this study investigated interrelations with pain duration, pain density, depression and cognitive performance, but relationship between the KOA grade, pain intensity, functional limitations, and quality of life were absent. Last, most patients have had long-term pain medication use. I acknowledge their impact could potentially have had influential effects on the results obtained.

## 6 Conclusion

We found whole brain GMV and regional GMV decrease occurs in KOA patients. The exact process underlying this gray matter loss remains obscure. Cell atrophy or synaptic loss as well as simple decreases in cell size or blood volume have been suggested as possible explanations. Neuroimaging studies identify a pain network mainly about S1, S2, IC, ACC, and PFC. In contrary to acute pain, chronic pain conditions preferentially involve PFC, which is implied that chronic pain conditions have stronger cognitive, emotional, and introspective components than acute pain conditions.^[22]^ chronic pain should be considered a “cognitive state.”^[53]^ In this study, a high proportion of KOA patients exerted abnormal scores for MMSE, MoCA, HAMD, and HAMA compare to controls. Emotion and cognitive function about chronic pain should be highly regarded. Some limitations in this study might have affected our results, and further well-designed studies and intensive study are required to confirm the study findings.

## 7 Author contributions

**Data curation:** X. Liao, C. Mao, Q. Zhang.

**Formal analysis:** X. Liao.

**Methodology:** C. Mao.

**Project administration:** C. Mao.

**Software:** C. Mao.

**Supervision:** M. Zhang, X. Yang.

**Writing – original draft:** X. Liao.

**Writing – review & editing:** Y. Wang, D. Cao, D.A. Seminowicz.
